# Allopurinol-Induced Leukocytoclastic Vasculitis With Systemic Inflammatory Response: A Case Report From Azerbaijan

**DOI:** 10.7759/cureus.113944

**Published:** 2026-08-04

**Authors:** Divya Ravikumar, Godly Silven, Hamida Salimzade

**Affiliations:** 1 Department of Allergy and Immunology, Azerbaijan Medical University, Baku, AZE

**Keywords:** allopurinol, drug-induced vasculitis, eosinophilia, hypersensitivity syndrome, leukocytoclastic vasculitis, plasmapheresis, systemic inflammation

## Abstract

Allopurinol is a commonly prescribed xanthine oxidase inhibitor used in the management of hyperuricemia and gout. Although generally well tolerated, it is associated with rare but potentially life-threatening hypersensitivity reactions, including allopurinol-induced vasculitis. We present the case of a 66-year-old woman who developed an acute cutaneous eruption 14 days after initiating allopurinol 100 mg daily for the management of recurrent gout. She subsequently developed systemic inflammatory manifestations, hepatic involvement, hematologic abnormalities, and immunologic activation consistent with drug-induced vasculitis following exposure to allopurinol. Laboratory findings demonstrated marked elevation of inflammatory markers, liver enzymes, immunoglobulin E levels, and evolving hematologic changes, including eosinophilia followed by lymphocytosis. The patient required plasmapheresis as part of management. This case highlights the importance of early recognition of drug-induced vasculitis and the need for prompt intervention to prevent progression to severe systemic complications.

## Introduction

Allopurinol is the first-line urate-lowering therapy for the management of gout and chronic hyperuricemia owing to its efficacy, affordability, and favorable long-term safety profile. Despite its widespread use, allopurinol remains one of the most common causes of severe cutaneous adverse reactions (SCARs), including allopurinol hypersensitivity syndrome (AHS), drug reaction with eosinophilia and systemic symptoms (DRESS), Stevens-Johnson syndrome (SJS), toxic epidermal necrolysis (TEN), and, less commonly, drug-induced leukocytoclastic vasculitis (LCV) [1]. Although the overall incidence of AHS is estimated to be only 0.1-0.4% among treated patients, mortality rates may exceed 20-25%, particularly in those with delayed diagnosis and multiorgan involvement [2].

LCV is an immune complex-mediated small-vessel vasculitis characterized histologically by neutrophilic infiltration of postcapillary venules, leukocytoclasia, fibrinoid necrosis, and erythrocyte extravasation [3]. Clinically, patients typically present with palpable purpura involving the dependent areas of the lower extremities; however, severe cases may demonstrate systemic manifestations involving the liver, kidneys, gastrointestinal tract, joints, and peripheral nervous system. Drug-induced LCV accounts for approximately 10-20% of all cases of cutaneous small-vessel vasculitis, with antibiotics, nonsteroidal anti-inflammatory drugs, and allopurinol representing well-recognized triggers [4].

The pathogenesis of allopurinol-induced hypersensitivity involves complex interactions between oxypurinol accumulation, T-cell-mediated immune activation, cytokine release, immune complex deposition, and genetic susceptibility, particularly in individuals carrying the HLA-B*58:01 allele [5]. Early withdrawal of the offending medication remains the cornerstone of management; however, severe systemic disease may require systemic corticosteroids and, in selected refractory cases, therapeutic plasma exchange to rapidly remove circulating inflammatory mediators and drug metabolites [1].

Herein, we present a clinically significant case of suspected allopurinol-induced LCV with systemic inflammatory response and acute hepatocellular injury that developed within two weeks of initiating allopurinol therapy. The patient demonstrated progressive clinical deterioration despite prompt discontinuation of the drug and initiation of high-dose corticosteroids but subsequently experienced rapid clinical recovery following therapeutic plasmapheresis. This case highlights the importance of early recognition, timely immunomodulatory therapy, and consideration of plasma exchange in severe refractory presentations.

## Case presentation

A 66-year-old woman presented to the emergency department with features suggestive of an acute allergic reaction. Fourteen days prior to admission, the patient was prescribed allopurinol 100 mg daily for the management of recurrent gout. She was taking amlodipine 5 mg daily for mild hypertension, a medication she had tolerated without incident for over five years. She was not taking any other routine medications or over-the-counter supplements.

Upon physical examination, the patient exhibited a widespread, non-blanching erythematous maculopapular rash with palpable purpura prominently distributed over her bilateral lower extremities and extending to her trunk (Figure 1). There was no initial evidence of pulmonary involvement on chest radiography, which was reported as normal. The patient was admitted with a clinical diagnosis of an acute hypersensitivity reaction, and allopurinol was immediately discontinued.

**Figure 1:**
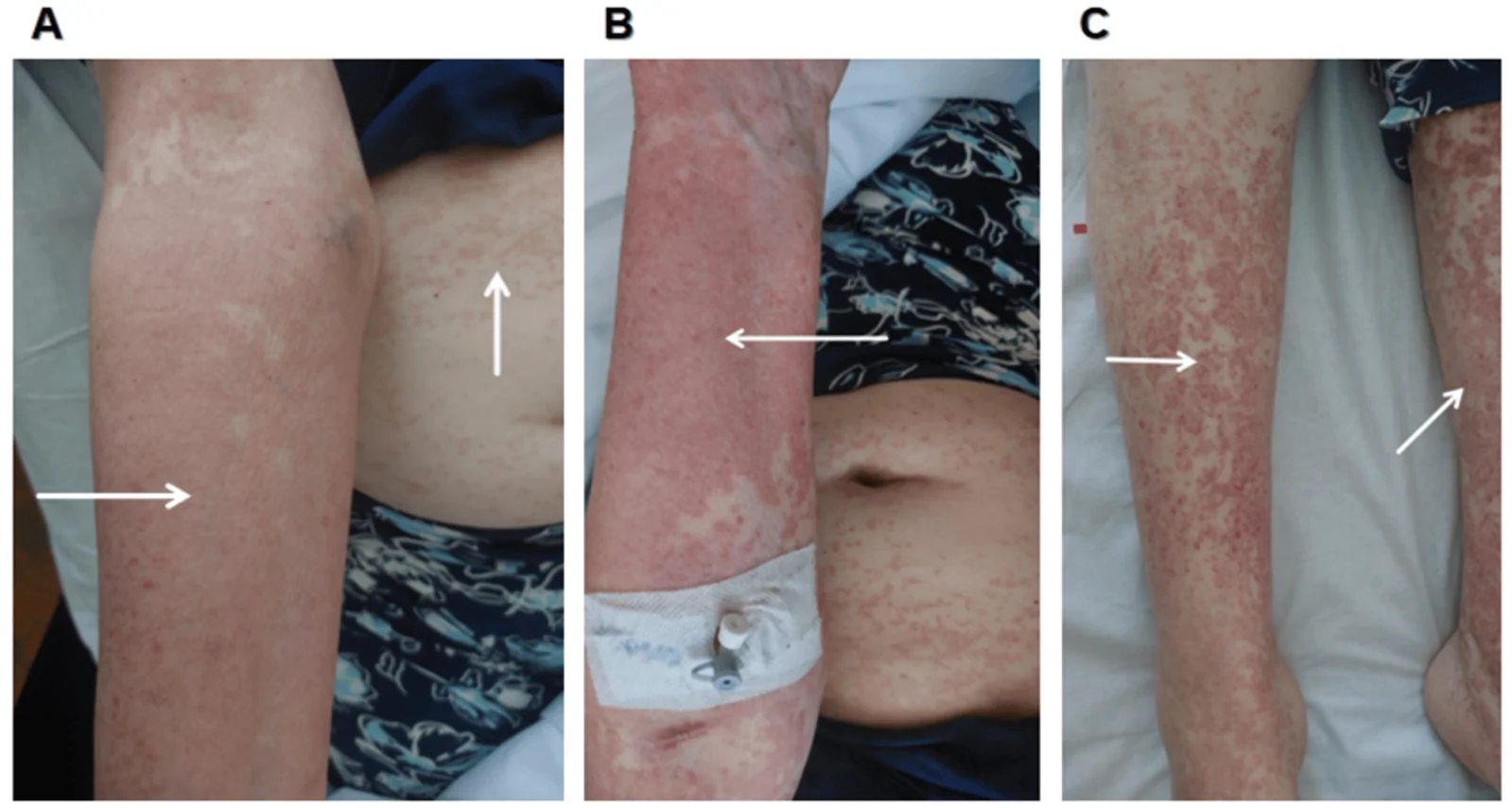
Clinical photograph demonstrating palpable purpura and erythematous maculopapular rash on the bilateral lower extremities (A) Upper extremity and trunk: the arrows highlight a scattered, erythematous maculopapular eruption on the abdomen and a fainter, diffuse erythema on the forearm. (B) Progression or more severe area of involvement on the upper extremity (with a peripheral IV cannula in situ): the arrow points to dense, coalescing erythema that is much more confluent than in A. (C) Bilateral lower extremities: the arrows point to the classic hallmark sign of leukocytoclastic vasculitis: a highly dense, non-blanching, purpuric maculopapular rash (palpable purpura) that is coalescing over the shins and calves.

Initial laboratory evaluation revealed eosinophilia (10.1%), elevated liver enzymes (alanine aminotransferase (ALT) 160 U/L and aspartate aminotransferase (AST) 85 U/L), and mildly elevated C-reactive protein (7 mg/L) (Table 1). Urinalysis demonstrated mild proteinuria, bilirubin positivity, and the presence of salt crystals and mucus, suggesting early systemic involvement.

**Table 1:** Laboratory trends WBC: white blood cell count; CRP: C-reactive protein; ALT: alanine aminotransferase; AST: aspartate aminotransferase; IgE: immunoglobulin E

Parameter	Day 1	Day 3	Reference value
WBC (×10^3^/mUL)	8.7	13.46 ↑	4.20-11.17
Eosinophils (%)	10.1 ↑	0.4	0.2-5.3
Lymphocytes (×10^3^/mUL)	1.52	5.36 ↑	0.90-3.77
CRP (mg/L)	7 ↑	82 ↑↑	<5
ALT (U/L)	160 ↑	385 ↑↑	<34
AST (U/L)	85 ↑	110 ↑	<32
IgE (IU/mL)	128.9 ↑	130.8 ↑	<100
D-dimer (ng/mL)	-	2210 ↑	<500

Subsequent laboratory evaluation three days later demonstrated significant clinical deterioration despite immediate drug withdrawal and initiation of high-dose systemic corticosteroids (intravenous methylprednisolone 1 mg/kg/day). The cutaneous lesions showed no clinical improvement even after the cessation of the offending medication (Figure 2).

**Figure 2:**
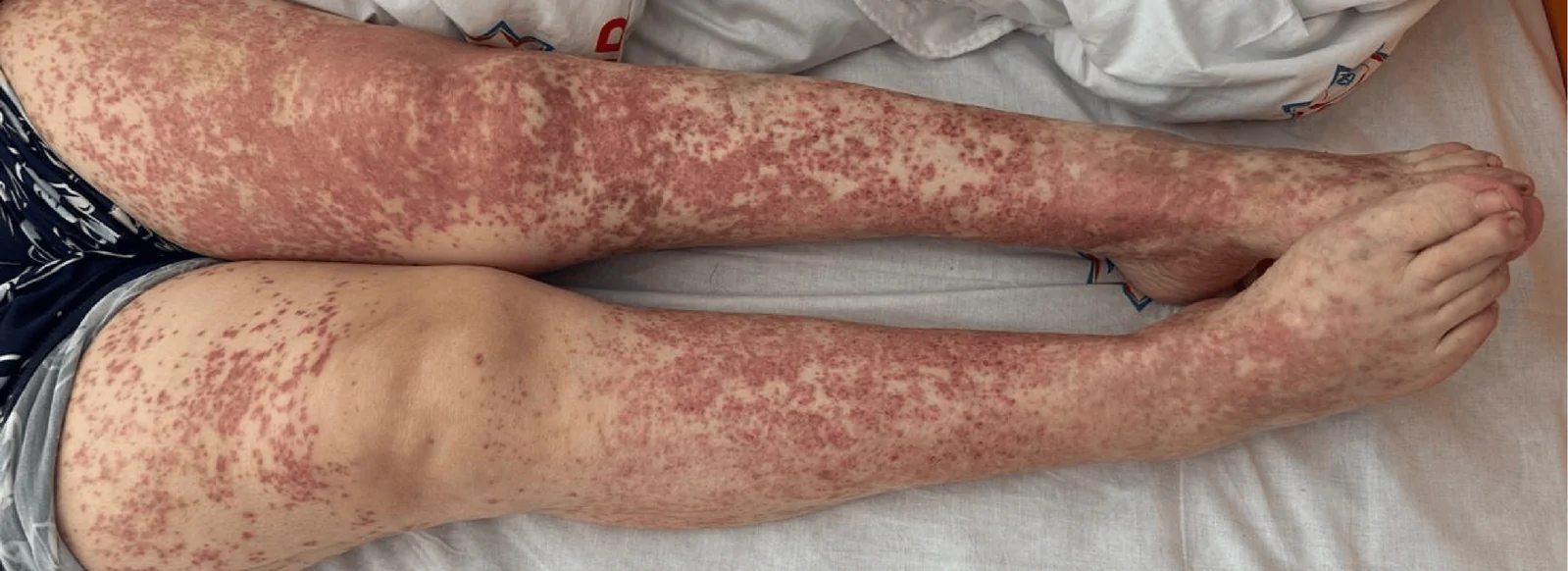
Persistence of the erythematous maculopapular rash and palpable purpura despite 72 hours of drug cessation and systemic corticosteroid therapy

There was marked leukocytosis (white blood cell count (WBC) 13.46×10³/µL) with lymphocytosis (5.36×10³/µL) and normalization of eosinophils, indicating a dynamic immune response. C-reactive protein increased significantly to 82 mg/L, reflecting a severe inflammatory state. Liver function tests showed substantial elevation (ALT 385 U/L and AST 110 U/L), consistent with acute hepatocellular injury.

Biochemical analysis revealed hypoproteinemia (total protein 48.9 g/L) and hypoalbuminemia (29.9 g/L), indicating systemic inflammation and possible capillary leak or hepatic dysfunction. Immunological testing showed elevated IgE levels (130.8 IU/mL), supporting an allergic or hypersensitivity-mediated process. Coagulation parameters remained within near-normal limits; however, D-dimer was markedly elevated (2210 ng/mL), suggesting the activation of the coagulation cascade and possible endothelial injury. Electrocardiographic findings demonstrated sinus rhythm with left ventricular hypertrophy, indicating possible systemic effects of inflammation.

Given the severity of systemic involvement and rapid clinical deterioration and worsening hepatic function despite 72 hours of intravenous corticosteroid therapy, the patient underwent therapeutic plasmapheresis. A total of 1750 mL of blood was processed, with 800 mL of plasma removed and replaced with crystalloid solutions. The procedure was well tolerated, with stable hemodynamic parameters throughout.

Following plasmapheresis, the patient demonstrated rapid and significant clinical improvement. Her cutaneous lesions began to fade, and her inflammatory markers and transaminases trended downward. She was discharged 10 days later, with a tapering dose of oral prednisone (starting at 60 mg daily and decreasing by 10 mg weekly). At her four-week outpatient follow-up, all laboratory parameters had normalized, and the purpuric rash had completely resolved with post-inflammatory hyperpigmentation (Figure 3).

**Figure 3:**
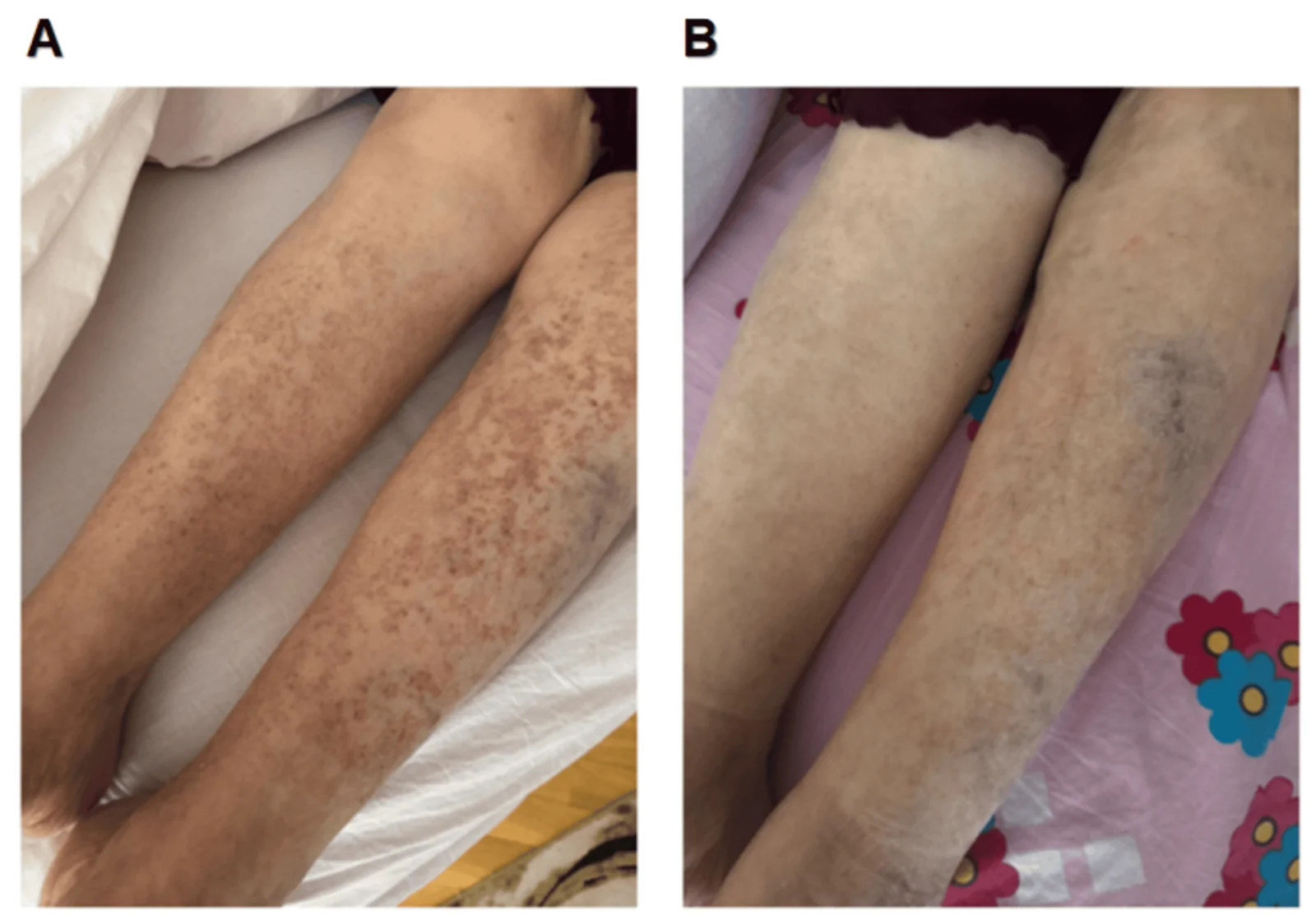
Complete clinical resolution of the purpuric rash on the bilateral lower extremities at the four-week outpatient follow-up, demonstrating expected post-inflammatory hyperpigmentation and an absence of active vasculitic lesions (A) Initial clinical improvement following therapeutic plasmapheresis, demonstrating the fading of active palpable purpura into sub-acute, non-inflammatory macules. (B) Complete clinical recovery and total clearance of the rashes at the final follow-up, with minimal residual post-inflammatory hyperpigmentation.

To objectively determine the probability of a drug-induced adverse reaction, the Naranjo Adverse Drug Reaction Probability Scale [6] was applied, demonstrating a probable causal association between allopurinol and the patient's clinical presentation (Table 2).

**Table 2:** Causality assessment (Naranjo Adverse Drug Reaction Probability Scale) Total score: 7 → probable adverse drug reaction

Question	Score
Previous conclusive reports on this reaction	+1
Event appeared after suspected drug	+2
Improvement after drug withdrawal	+1
Alternative causes unlikely	+2
Objective evidence present	+1

Although the patient demonstrated several features suggestive of DRESS, including eosinophilia, hepatic involvement, and extensive cutaneous eruption, she did not fulfill the complete RegiSCAR diagnostic criteria [7] because fever, lymphadenopathy, and atypical lymphocytosis were absent or not documented (Table 3). Consequently, the clinical presentation was considered more consistent with allopurinol-induced LCV occurring within the broader spectrum of AHS.

**Table 3:** RegiSCAR scoring system for DRESS syndrome applied to the present case Total RegiSCAR score: 5 The overlap between drug-induced vasculitis and DRESS has been recognized in the literature, and these entities may coexist in severe hypersensitivity reactions. DRESS: drug reaction with eosinophilia and systemic symptoms; ALT: alanine aminotransferase; AST: aspartate aminotransferase

RegiSCAR criterion	Present case	Score
Fever ≥38.5°C	Absent	0
Enlarged lymph nodes involving ≥2 sites	Absent	0
Eosinophilia	Present (10.1%)	+1
Atypical lymphocytes	Not reported	0
Skin rash >50% body surface area	Present	+1
Rash suggestive of DRESS (infiltrative/purpuric)	Present	+1
Skin biopsy suggestive of DRESS	Not performed	0
Internal organ involvement	Liver (ALT/AST elevation)	+1
Resolution ≥15 days	Clinical improvement with treatment	0*
Evaluation of other potential causes	Alternative diagnoses reasonably excluded	+1

## Discussion

Drug-induced LCV represents an uncommon but important manifestation of allopurinol hypersensitivity [1-3]. Although cutaneous vasculitis generally remains confined to the skin, severe cases may progress to systemic involvement affecting the liver, kidneys, gastrointestinal tract, and other organs [8]. Recognition of this rare presentation is essential because delayed diagnosis may result in significant morbidity and mortality [9].

The chronological evolution observed in this patient strongly supports an immune-mediated hypersensitivity reaction. The development of palpable purpura 14 days after the initiation of allopurinol corresponds closely with the typical latency period reported for drug-induced LCV and AHS. Initial eosinophilia followed by marked lymphocytosis likely reflects the dynamic transition from an early eosinophil-mediated allergic response toward subsequent T-cell activation and systemic immune dysregulation. Concurrent elevation of serum IgE further supports the activation of a type IV hypersensitivity pathway.

The progressive increase in inflammatory markers despite prompt discontinuation of allopurinol illustrates the persistent immune activation that characterizes severe drug hypersensitivity reactions. Similarly, the marked elevation of ALT and AST indicates significant hepatocellular injury, one of the most frequently affected internal organs in AHS. The presence of hypoalbuminemia and elevated D-dimer further suggests widespread endothelial injury, increased vascular permeability, and activation of coagulation pathways secondary to systemic inflammation. To place the present case in the context of existing evidence, a summary of recent studies on allopurinol-induced LCV, AHS, and related SCARs is presented in Table 4.

**Table 4:** Summary of published literature on allopurinol-induced leukocytoclastic vasculitis and allopurinol hypersensitivity syndrome DRESS: drug reaction with eosinophilia and systemic symptoms; DIHS: drug-induced hypersensitivity syndrome; ANCA: antineutrophil cytoplasmic antibody

Study	Study design	Study population	Main findings	Clinical relevance to the present case
Hama et al., 2022 [4]	Comprehensive review	DIHS/DRESS literature	Reviewed immunopathogenesis, T-cell activation, cytokine dysregulation, eosinophilia, viral reactivation, and multiorgan involvement in DRESS. Mortality ~10%	Supports the immune-mediated mechanism underlying eosinophilia, hepatic injury, systemic inflammation, and progression observed in the present patient
Liu et al., 2022 [1]	Retrospective case series	52 patients with allopurinol-induced DRESS	Hepatic involvement was the most frequent visceral manifestation. Early drug withdrawal and corticosteroids improved outcomes; delayed recognition increased morbidity	Reinforces the importance of prompt diagnosis and corticosteroid therapy in patients with liver involvement after allopurinol exposure
Liu and Zhao, 2022 [2]	Clinical case series	46 patients with allopurinol-induced DRESS	Demonstrated typical latency of 2-6 weeks after allopurinol initiation, frequent eosinophilia, liver dysfunction, and systemic inflammatory manifestations	Mirrors the 14-day onset, eosinophilia, and hepatocellular injury observed in the present case
Yaseen et al., 2023 [3]	Clinical review	Allopurinol hypersensitivity syndrome	Reported an incidence of approximately one per 1,000 allopurinol users with mortality approaching 20-25%. Highlighted the importance of HLA-B*58:01 screening in high-risk populations and immediate drug discontinuation	Provides epidemiologic context and supports early recognition and withdrawal of allopurinol in suspected allopurinol hypersensitivity syndrome
Calle et al., 2023 [6]	Narrative review	DRESS syndrome literature	Proposed a practical diagnostic and treatment algorithm emphasizing RegiSCAR criteria, corticosteroids, and multidisciplinary management	Supports inclusion of the RegiSCAR assessment and differentiation of DRESS from leukocytoclastic vasculitis
Walsh et al., 2022 [8]	Systematic review and meta-analysis	Patients with ANCA-associated vasculitis undergoing plasma exchange	Evaluated the role of therapeutic plasma exchange in severe immune-mediated vasculitis, demonstrating benefit in selected high-risk patients while emphasizing individualized decision-making	Provides mechanistic justification for plasmapheresis as rescue therapy in severe immune-mediated disease, although evidence is indirect for drug-induced leukocytoclastic vasculitis
de Groot, 2022 [9]	Review articles	Cutaneous small-vessel vasculitis	Summarized the diagnosis, histopathology, differential diagnosis, treatment, and prognosis of cutaneous leukocytoclastic vasculitis. Emphasized skin biopsy as the diagnostic gold standard	Supports discussion of histopathology, explains why biopsy is recommended, and helps justify the diagnostic approach in the absence of biopsy

Compared with previously published reports, our patient demonstrated a characteristic latency period of 14 days following the initiation of allopurinol, followed by rapidly progressive palpable purpura, eosinophilia, hepatocellular injury, and marked systemic inflammation. While these clinical features overlap with the spectrum of AHS and DRESS, the predominant vasculitic presentation, absence of fever and lymphadenopathy, and favorable response to corticosteroids combined with therapeutic plasmapheresis distinguish this case. Notably, reports describing successful plasmapheresis in suspected allopurinol-induced LCV remain limited, highlighting the educational value and clinical relevance of the present report [9,10].

Histopathological confirmation through skin biopsy remains the diagnostic gold standard for LCV and typically demonstrates neutrophilic infiltration of postcapillary venules, leukocytoclasia, fibrinoid necrosis, immune complex deposition, and erythrocyte extravasation [10]. Although biopsy was not performed in the present case because of the patient's rapidly progressive clinical deterioration and the need for urgent therapeutic intervention, the diagnosis was supported by the characteristic clinical presentation, temporal association with allopurinol initiation, exclusion of competing diagnoses, favorable Naranjo causality assessment, and complete clinical recovery following the withdrawal of allopurinol and immunomodulatory treatment. Given the overlapping clinical features with several inflammatory, autoimmune, infectious, and drug-induced conditions, the principal differential diagnoses considered in the present case and the rationale for their exclusion are summarized in Table 5.

**Table 5:** Differential diagnosis considered in the present case of suspected allopurinol-induced leukocytoclastic vasculitis and rationale for exclusion DRESS: drug reaction with eosinophilia and systemic symptoms; ESR: erythrocyte sedimentation rate; CRP: C-reactive protein; RBC: red blood cell; WBC: white blood cell; ANCA: antineutrophil cytoplasmic antibody

Condition	Key clinical features	Laboratory findings	Histopathology	Distinguishing features from this case	Reason for exclusion
Drug-induced leukocytoclastic vasculitis	Palpable purpura, erythematous maculopapular rash, pruritus; onset days to weeks after drug exposure	Elevated ESR/CRP; may show mild leukocytosis ± eosinophilia	Neutrophilic infiltration, leukocytoclasia, fibrinoid necrosis, RBC extravasation	Temporal association with drug; biopsy confirms small-vessel vasculitis	Confirmed diagnosis based on a 14-day timeline, classic clinical presentation, and clinical course (biopsy deferred due to acuity)
DRESS syndrome	Rash, fever, facial edema, lymphadenopathy, multiorgan involvement (liver, kidney)	Marked eosinophilia, atypical lymphocytes, elevated liver enzymes	Interface dermatitis with eosinophils	Systemic involvement and organ dysfunction prominent	While hepatic injury was evident, a definitive diagnosis of DRESS syndrome was precluded by the absence of other obligatory criteria, specifically fever, lymphadenopathy, and sustained eosinophilia
Stevens-Johnson syndrome/toxic epidermal necrolysis	Painful rash, mucosal involvement, epidermal detachment, targetoid lesions	Elevated inflammatory markers; possible electrolyte imbalance	Full-thickness epidermal necrosis	Prominent mucosal lesions and skin detachment	No mucosal involvement or epidermal necrolysis
Atopic dermatitis	Chronic pruritic eczema, flexural involvement, personal/family history of atopy	Usually normal; elevated IgE possible	Spongiosis, epidermal hyperplasia	Chronic relapsing course, typical distribution	Acute onset, widespread distribution, biopsy inconsistent
Allergic contact dermatitis	Localized erythematous rash at the site of allergen exposure	Typically normal	Spongiotic dermatitis	Clear exposure to external allergen; localized lesions	No identifiable contact trigger; generalized distribution
Infectious exanthema	Diffuse rash, often with fever and systemic symptoms	Elevated WBC, infection markers	Variable; often non-specific	Associated with infection (viral/bacterial)	No fever or infectious symptoms
Primary systemic vasculitis (e.g., ANCA-associated)	Purpura with systemic involvement (renal, pulmonary, neurological)	Positive ANCA, abnormal renal parameters	Necrotizing vasculitis	Multisystem involvement	Negative ANCA, no systemic signs
Urticaria	Transient wheals, intense itching, lesions resolve within 24 hours	Usually normal	Dermal edema without vasculitis	Lesions are fleeting, not persistent	Lesions persistent and progressive
Senile xerosis with pruritus	Dry skin, mild itching in elderly	Normal	Non-inflammatory changes	No inflammatory rash or systemic features	Does not explain rash progression or biopsy findings

Although several differential diagnoses were considered, the close temporal relationship with allopurinol exposure, characteristic palpable purpura, eosinophilia, hepatic involvement, elevated inflammatory markers, and complete recovery following drug withdrawal strongly favored allopurinol-induced LCV. The absence of fever, mucosal involvement, lymphadenopathy, and positive autoimmune serology further reduced the likelihood of alternative diagnoses.

Differentiating LCV from DRESS syndrome can be challenging because both conditions may present with cutaneous eruptions, eosinophilia, and multiorgan involvement [11,12]. However, unlike classic DRESS syndrome, our patient lacked fever, lymphadenopathy, and persistent eosinophilia while demonstrating predominant palpable purpura compatible with small-vessel vasculitis. Similarly, the absence of mucosal involvement, epidermal detachment, and targetoid lesions effectively excluded SJS and TEN.

An important feature of this case was the successful use of therapeutic plasmapheresis following inadequate clinical response to high-dose systemic corticosteroids [13-15]. Plasma exchange has been proposed as rescue therapy in severe drug-induced hypersensitivity reactions by facilitating the rapid removal of circulating drug metabolites, immune complexes, cytokines, and pathogenic antibodies responsible for ongoing endothelial injury [8]. Although evidence supporting plasmapheresis remains limited to case reports and small case series, the dramatic clinical and biochemical improvement observed in our patient supports its consideration in carefully selected patients with severe refractory systemic disease.

The principal limitation of this report is the absence of histopathological confirmation by skin biopsy, which remains the gold standard for diagnosing LCV. Additionally, HLA-B*58:01 genotyping and a complete RegiSCAR assessment were not available, limiting the definitive characterization of the hypersensitivity syndrome. Nevertheless, the strong temporal relationship with allopurinol exposure, characteristic clinical findings, supportive laboratory abnormalities, exclusion of alternative diagnoses, probable Naranjo causality score, and complete recovery following drug withdrawal and immunomodulatory therapy strongly support the diagnosis of allopurinol-induced LCV with systemic involvement.

This case also emphasizes the importance of recognizing risk factors for allopurinol hypersensitivity. Previous studies have identified advanced age, renal impairment, high starting doses of allopurinol, concomitant diuretic therapy, and carriage of the HLA-B*58:01 allele as important predisposing factors. Increasing implementation of pharmacogenetic screening in high-risk populations may significantly reduce the incidence of severe allopurinol-associated adverse reactions.

## Conclusions

This case highlights a rare presentation of suspected allopurinol-induced LCV with systemic inflammatory response and acute hepatocellular injury occurring within the spectrum of AHS. Early recognition of evolving hypersensitivity reactions, prompt discontinuation of the offending medication, and timely initiation of immunomodulatory therapy are essential to prevent irreversible multiorgan damage. The favorable clinical response following therapeutic plasmapheresis suggests that plasma exchange may represent an effective rescue strategy in selected patients with severe corticosteroid-refractory disease. Increased clinician awareness of this uncommon presentation may facilitate earlier diagnosis and improve patient outcomes.
